# Development of Follicle-Stimulating Hormone Receptor Binding Probes to Image Ovarian Xenografts

**DOI:** 10.4172/2155-952X.1000198

**Published:** 2015-09-10

**Authors:** Chung-Wein Lee, Lili Guo, Daniela Matei, Keith Stantz

**Affiliations:** 1 Medical Physics Program, School of Health Science, Purdue University, West Lafayette, IN, USA; 2 Department of Medicine, Indiana University School of Medicine, Indianapolis IN, USA

**Keywords:** Ovarian cancer, Follicle-stimulating hormone receptor, Decapeptide, Multimeric peptide, Near-infrared imaging

## Abstract

The Follicle-Stimulating Hormone Receptor (FSHR) is used as an imaging biomarker for the detection of ovarian cancer (OC). FSHR is highly expressed on ovarian tumors and involved with cancer development and metastatic signaling pathways. A decapeptide specific to the FSHR extracellular domain is synthesized and conjugated to fluorescent dyes to image OC cells *in vitro* and tumors xenograft model *in vivo*. The *in vitro* binding curve and the average number of FSHR per cell are obtained for OVCAR-3 cells by a high resolution flow cytometer. For the decapeptide, the measured EC50 was 160 μM and the average number of receptors per cell was 1.7 × 10^7^. The decapeptide molecular imaging probe reached a maximum tumor to muscle ratio five hours after intravenous injection and a dose-dependent plateau after 24–48 hours. These results indicate the potential application of a small molecular weight imaging probe specific to ovarian cancer through binding to FSHR. Based on these results, multimeric constructs are being developed to optimize binding to ovarian cells and tumors.

## Introduction

Ovarian cancer (OC) is the leading cause of mortality from gynecological cancer in women, with a five year survival rate less than 45% [1,2]. OC occurs commonly in postmenopausal women. Due to lack of specific symptoms and reliable screening procedures, the majority of women with OC (60–65% of patients) are diagnosed late (stages III-IV) when the cancer has spread beyond the ovaries, resulting in a 5 year survival rate of 16–28% [3]. Several hypotheses to explain the etiology of ovarian epithelial cancer have been proposed, including the incessant ovulation hypothesis [4,5], the gonadotropin theory [6,7], and the sex-steroid hormones hypothesis [8,9]. Current epidemiologic studies, and models of OC, do not provide a satisfactory explanation for ovarian carcinogenesis [3,10]. Overall the molecular mechanisms of ovarian epithelial cancer tumorigenesis and metastasis remain unclear [3,10,11]. Searching novel diagnostic markers to improve early ovarian cancer detection has been an active research [12,13].

Some institutions perform annual pelvic exam to screen early ovarian tumor. Transvaginal ultrasound and serial measurements of CA-125 have included for high-risk population where CA125 is a membrane associated mucin on the surface of epithelial cells of ovarian cancer that is released within the blood and used to screen for ovarian cancer. The sensitivity of CA-125 in detecting early ovarian cancer is less than 60% [14,15]. Even together with ultrasound screening, the positive predictive value improves only about 20% [16,17]. Utilizing short peptide to receptor specific recognition in nuclear medicine has shown success in some cancer imaging. ^111^In labeled DTPAoctreotide (OctreoScan) binding to somatostatin receptors on cell surface approved by FDA has proven to be a successful and versatile imaging agent for primary and metastatic neuroendocrine tumors [18–20]. Other peptides including Bombesin to target gastrin releasing peptide receptor, vasoactive ntestinal peptide (VIP) to target VIP receptor, Exendin to target glucagon-like peptide 1 (GLP-1) receptor, and RGD to target integrin are currently in development or in clinical trials.

Recent studies show that the Follicle Stimulating Hormone Receptor (FSHR) is highly expressed on ovarian epithelial cancer cells and facilitates the development and the progression of OC [21–25]. FSHR belongs to G protein-coupled receptor and consists of a transmembrane and an extracellular domain, where the former is characterized by a seven helical repeats and the latter by multiple leucine-rich repeats. FSHR is expressed on normal ovarian epithelium, e.g., the fallopian tubal epithelium, and shares about 70% transmembrane domain sequence with other G protein-coupled receptors, such as Luteinizing Hormone Receptor [22,26]. In normal epithelial tissue, FSH binds to FSHR and works synergistically with other steroid hormones to regulate follicular growth during the ovulation process. In OC, FSHR signaling influences the expression of important oncogenes such as MAPK, EGFR, c-myc, and HER-2/neu [21]. And, FSHR expression increases in the following order of disease progression: ovarian epithelial inclusions (OEIs), benign ovarian epithelial tumor (OET), and borderline OETs [3,27]. In some studies, it has been reported that FSHR level can decrease between borderline OETs and ovarian carcinomas [3,24]. This differential expression of FSHR allows the possibility to image OEC in different stages of formation based on the binding molecules to FSHR. To image FSHR *in vivo*, a hydrophilic deca amino acid sequence (BI-10) derived from human FSH binding inhibitor (~57 kDa) in human follicular fluid [28,29] identified by DW Lee et al. is employed. A functional radio ligand receptor assay shows that the synthetic BI-10 peptide specifically binds to FSHR and inhibits FSH-stimulated estradiol and secondary messenger cAMP synthesis at high concentrations, where the possible BI-10 binding site were shown to overlap with the FSH-β unit [30].

In this study, the goal is to determine the feasibility of imaging ovarian tumors based on the binding of BI-10 to FSHR *in vitro* and *in vivo*. First, the BI-10 deca peptide was synthesized. A fluorescein was conjugated to its N-terminal to quantify the *in vtiro* binding affinity to ovarian epithelial cancer cells using flow cytometry techniques. Next, a near-infrared fluorochrome was conjugated to the BI-10 and tested *in vivo* by optical imaging in subcutaneous ovarian tumors in xenograft mouse model. This was used to determine the optimal set of imaging protocols based on the pharmacokinetics of the BI-10 molecular imaging probe to determine the time after injection when the tumor-to-reference tissue ratio (TRR) is at a maximum and the reproducibility (or error) in these measurements across a cohort of mice.

## Material and methods

### Synthesis of BI-10 peptide

FSH receptor-binding inhibitor fragment (BI-10), TENLEPNGEGNH_2_ was synthesized by standard solid-phase Fmoc chemistry on an ABI 433 peptide synthesizer, starting with Fmoc-amide resin. Side-chain protecting groups were Trt for Asn, tBu for Thr and OtBu for Glu. Once peptide chain assembly was complete, the peptide was cleaved from the resin using TFA cocktail reagent (TFA: phenol: water: thioanisol: TIPS, 100:5:5:2.5). 10 ml of cleavage solution was added to 400 mg of resin and allowed to shake for two hours. The resin was filtered, and the peptide was precipitated by excess cold ether. The crude peptide was purified by C_18_-RP HPLC (Vydac 218TP10155) and identified by analytical HPLC and MALDI mass spectroscopy.

### Conjugation of fluorescein to BI-10 decapeptide (BI-10^FAM^) molecular probe

5(6)-FAM, SE [5-(and-6)-carboxyfluorescein, succinimidyl ester, Anapec) was conjugated to the N-terminus of the peptide at room temperature in DMSO with a 2 to 1 molar equiv of 5(6)-FAM, SE to peptide, in the presence of 10 equiv of DIEA. The crude fluorescent-peptide was purified by C_18_-RP HPLC (Waters SunFire prep column, 5 μm, 30 × 150 mm). The final product was characterized by analytical HPLC and MALDI mass spectroscopy for purity (>95%) and composition.

### Preparation of OVCAR-3 cells for flow cytometry

OVCAR-3 cells (generously provided by Dr. Mellisa Fishell of Indiana University School of Medicine) were chosen for *in vitro* binding experiment due to its high level of FSHR expression [21]. For the first 2–3 passages, the cells were plated in 75 mm^2^ flasks using RPMI medium (supplemented with 5 μM Insulin (Sigma), 20% FBS (Hyclone), Sodium private (GIBCO), and Pen Strep (GIBCO)), and placed in an incubator (5% CO_2_ at a temperature of 37°C incubator) to maintain viability. For peptide binding experiments, cells were plated in six-well plates with the medium and environment conditions as previously described. Medium was changed every 2–3 days until cells reached more than 90% confluency. Prior to BI-10 peptide incubation, the medium was aspirated slowly to maintain OVCAR-3 cell sheet adhering to each well. Cells in each well were rinsed with 1 mL PBS (without calcium and magnesium ions) followed by slow aspiration. Five of the six wells were dispensed into 1 mL solution of the medium and different dilutions of the fluorescein conjugated BI-10 peptide: 1.0 μM, 10.0 μM, 50 μM, 100 μM, 250 μM, and 500 μM. The last well was dispensed into a 1 mL medium without peptides to act as control. The six-well plate was placed in the incubator for 30 minutes, after which, the medium was aspirated and each well was gently rinsed using PBS to remove any unbound peptides. To remove the OC cells from the plates and to maintain the integrity of the ectodomain of FSHR, the cells were exposed to 5 mM of EDTA in PBS. These cells were transferred to flow cytometry tubes and spun down at 1200 rpm for five minutes, after which the supernatant was replaced with fresh PBS buffer. The cell pellets were resuspended using a vortexer, replaced with fresh PBS buffer, and centrifuged a second time to remove any unbound peptides. These samples were ready for imaging within the flow cytometer. This procedure was repeated for each concentration of peptides three times.

### *In vitro* flow cytometry of BI-10^FAM^ binding affinity and receptor density

Each sample of OVCAR-3 cells was placed within the flow cytometer (Beckman Coulter FC500) and the FITC fluorescent intensity from the BI-10^FAM^ peptide bound to the OVCAR-3 cells were acquired. A TOPO-3 dye (Sigma) in 1 nM concentration was used to acquire the fluorescence from only viable cells in a two-color flow cytometry assay. Ten thousand cells were counted and the fluorescence measured in each sample. The median fluorescent intensity was plotted as a function of the log base 10 of the BI-10 concentration. The EC50 value (binding affinity) and receptor saturation intensity were calculated using a nonlinear regression analysis using GraphPad Prism fitting algorithm (GraphPad Software, Inc.). Each data point (concentration) is the average of three measurements. To calibrate the flow cytometer, the raw flow cytometry intensity from five different calibrated FITC fluorescence intensity micro beads (Quantum FITC MESF High Level beads, Bangs Labs, Fishers, IN) were measured under identical instrument settings, thus allowing the measured fluorescent intensities to be converted to a quantifiable number of BI-10 peptides. The QuickCal software (Bangs Labs) was used to obtain a semi-log of MESF, the Molecules of Equivalent Soluble Fluorochrome of calibration microspheres, versus flow cytometry fluorescent intensity. From this curve, the total number of receptors per OVCAR-3 cell was determined.

### Synthesis of a NIR FSH molecular imaging probe (BI-10^AF750^)

BI-10 peptide synthesis procedures were identical to those mentioned in the previous section. The near infrared dye, Alexa Fluor 750 (Invitrogen), was conjugated to the N-terminus of the BI-10 peptide. This was done at room temperature in DMSO with a one-to-one molar equivalent of Alexa Fluor 750 to peptide in the presence of 10 equivalent of triethylamine (Et_3_N). The conjugating reaction was monitored by analytical HPLC and was completed in 24 hours. The crude Alexa Fluor 750-peptide conjugate (BI-10^AF750^) was purified by C_18_-RP HPLC (Vydac 218-TP510). The purity of final product was checked by analytical HPLC to be in excess of 95%, and confirmation of the molecular mass was determined by MALDI mass spectroscopy (MNa^+^ 1946.8Da, found 1947.1Da).

### Animal model

All animal preparation and procedures for this study were reviewed and approved by the Indiana University School of Medicine Institutional Animal Care and Use Committee. Six mice with subcutaneous ovarian tumors were used in this study. Approximately 10^6^ SKOV3x ovarian cancer cells (0.1 mL PBS) were subcutaneously injected into the flank of (6–8 week) of each athymic nude mice (Harlan, Indianapolis, IN) and allowed to grow to a diameter of 8–10 mm (4–6 weeks). To determine the pharmacokinetics of BI-10^AF750^ molecular imaging probe and the approximate dose, the first group of three mice was each given a different dose of 120 μg, 40 μg, and 15 μg of the BI-10^AF750^ and its tumor-to-reference tissue (TRR) measured as a function of time. From this data, the time after injection that provides an optimal TRR will be determined. For the second cohort (n=3), each mouse will be given the same dose of BI-10^AF750^ (80 μg) to test the reproducibility of (error in) TRR *in vivo* imaging.

### *In Vivo* near-infrared fluorescence (NIRF) imaging

Prior to imaging, each mouse was anesthetized using a mixture of acepromazine (0.1 mg/kg, i.m.) and torbugesic (0.1 mg/kg, i.m.). A 27½ gauge butterfly catheter (Abbott Laboratories) flushed with heparin was inserted in the tail vein and secured. The catheter was connected to a syringe filled with BI-10^AF750^ peptide in PBS, which was mounted on a programmable infusion pump (BS-8000; Braintree Scientific, Inc.). The mouse and infusion pump was positioned within the light-tight box of the optical imager (LB981 NightOWL, Berthold) used for the measurement of the NIR emitting molecular Alexa Fluor 750 (Invitrogen). The excitation source was filtered using a HQ710/75X bandpass filter (Chroma Technologies Corp.) and uniformly illuminated the field of view of the mouse. The emission spectrum was filtered using a HQ810/90M bandpass filter to enhance the Alexa Fluoro 750 fluorescence relative to the auto fluorescence signal. Optical images were acquired prior to injection, every 3 seconds during the injection of the probe (50 μL/min), and every 60 seconds over the next 60–90 minutes using a 10 ms exposure time. Additional images were taken 4, 8, 12, 24 and 48 hours post-injection for the mice in the first group, while images of the mice in the second group were acquired at 6 hours post-injection using a 40 msec exposure time.

### Analysis of *in vivo* NIRF data

The tumor-to-reference tissue ratio (TRR) was calculated at each time point to assess the relative binding and uptake kinetics of the BI-10^AF750^ probe in ovarian tumors. To calculate the signal from the tumor, an ROI was drawn around the tumor and the mean (± sem) fluorescence signal divided by the exposure time was calculated (photons/mm^2^/s). The average signal (± sem) from the reference tissues was calculated from three ROIs placed within the hind-leg skeletal muscle, the forearm skeletal muscle, and the liver. For both groups of mice, the TRR was plotted as a function of time.

## Results

### Binding affinity of BI-10 decapeptide

**Figure 1:**
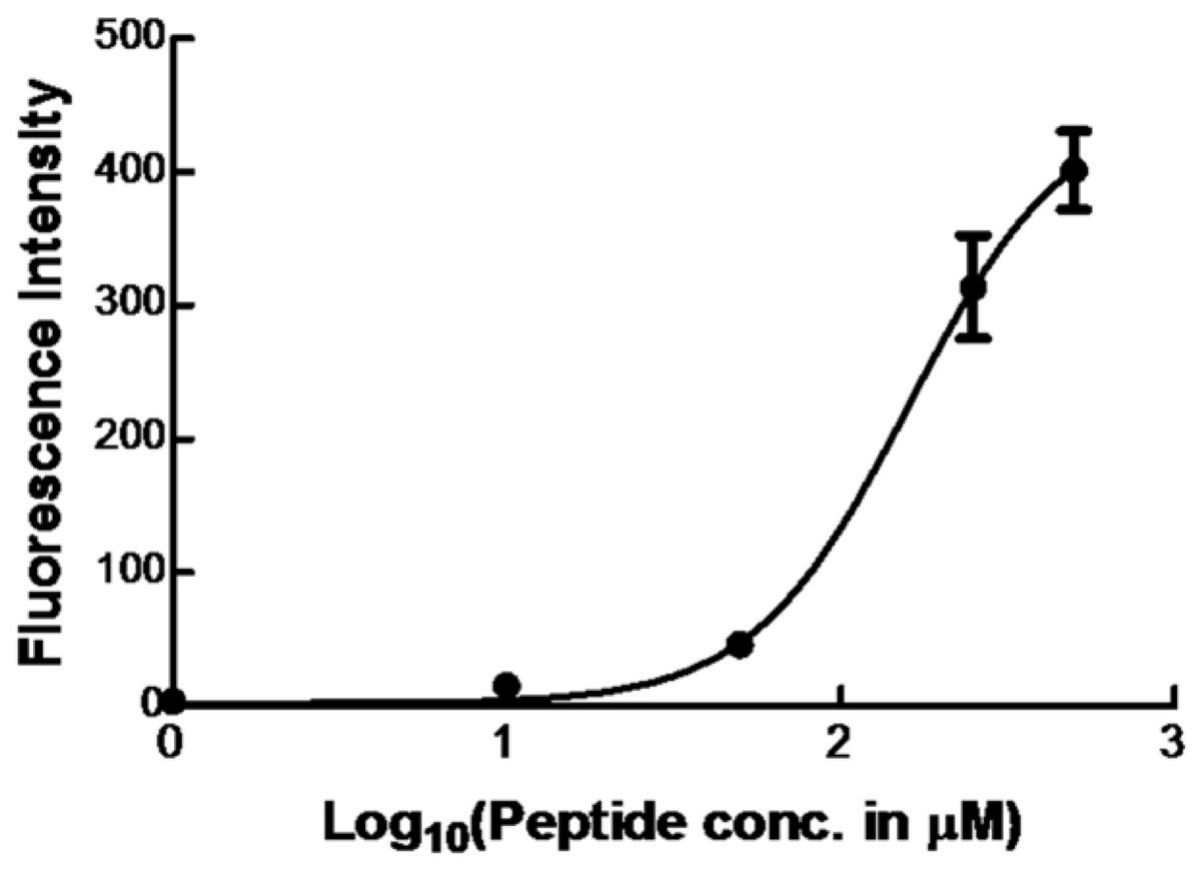
Dose Response Curve for BI-10^FAM^. The average fluorescence intensity and its standard deviation (n=5) was plotted as a function of peptide concentration (1.0 μM, 10.0 μM, 50 μM, 250 μM, and 500 μM) and fitted to a sigmoidal curve (solid line). Note, the error bars of the first three points are small and covered by the size of markers.

Flow cytometry is widely used to measure ligand binding to receptors expressed on cell membranes [31,32]. In Figure 1, the binding curve of the BI-10^FAM^ ligand is plotted. From this data, the EC50 of BI-10 was measured to be 160 μM (R^2^=0.93) and the saturation concentration was 449.5 μM, where the 95% confidence interval ranged from 337 to 561 μM. Based on this latter value, the number of FSH receptors per OVCAR-3 cell was estimated to be 1.7 × 10^7^. An important factor that could influence these results is if there is a loss of receptor function due to a significant amount of freely suspended fluorescein remaining in the buffer. To address this concern, cells were visually inspected prior to and after incubation with BI-10 peptides to assure cells were attached to the plate. Flow cytometry measurements were taken before and after numerous washes to assure unbound peptide were removed and stability of the bound peptide (see supplemental data), where measurements after the second wash was comparable to those after the third wash. Therefore, the data after second wash was used in all binding measurements from different peptide concentrations.

### *In vivo* kinetics and uptake

**Figure 2:**
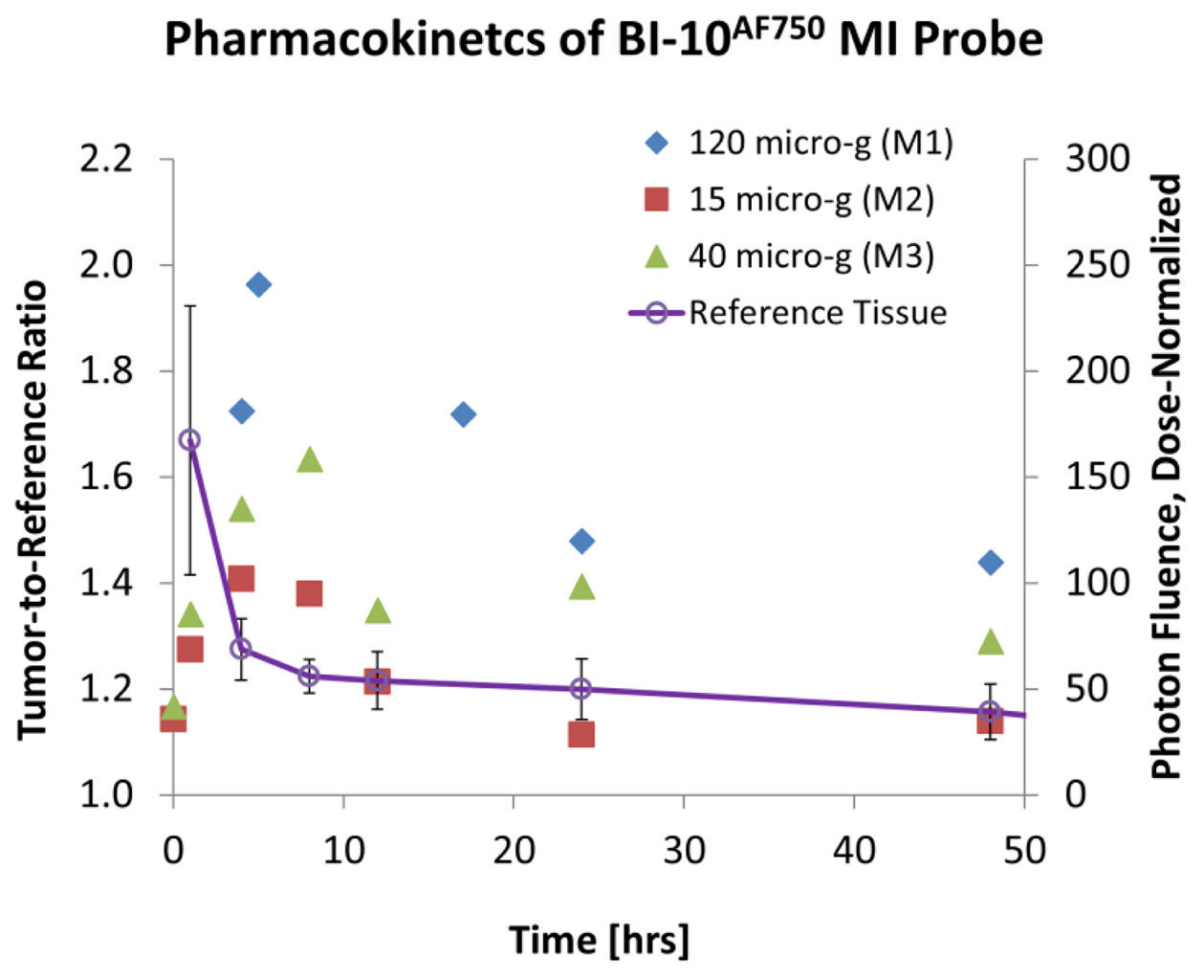
Kinematics of BI-10^AF750^ Molecular Imaging (MI) Probe. (*Primary Axis*) Plotted is the tumor-to-reference tissue (muscle) ratio (TRR) as a function of time for three mice (M1-M3) each injected with 120 μg (blue), 40 μg (green), and 15 μg (red) of the BI-10^AF750^ MI probe. The peak TRR occurred within 4–8 hours post-injection. (*Secondary Axis*) Similarly, the average dose-normalized fluorescence signal is plotted as function of time for muscle (n=3) from which the BI-10^AF750^ clearance rate was determined to be 4 hours.

**Figure 3:**
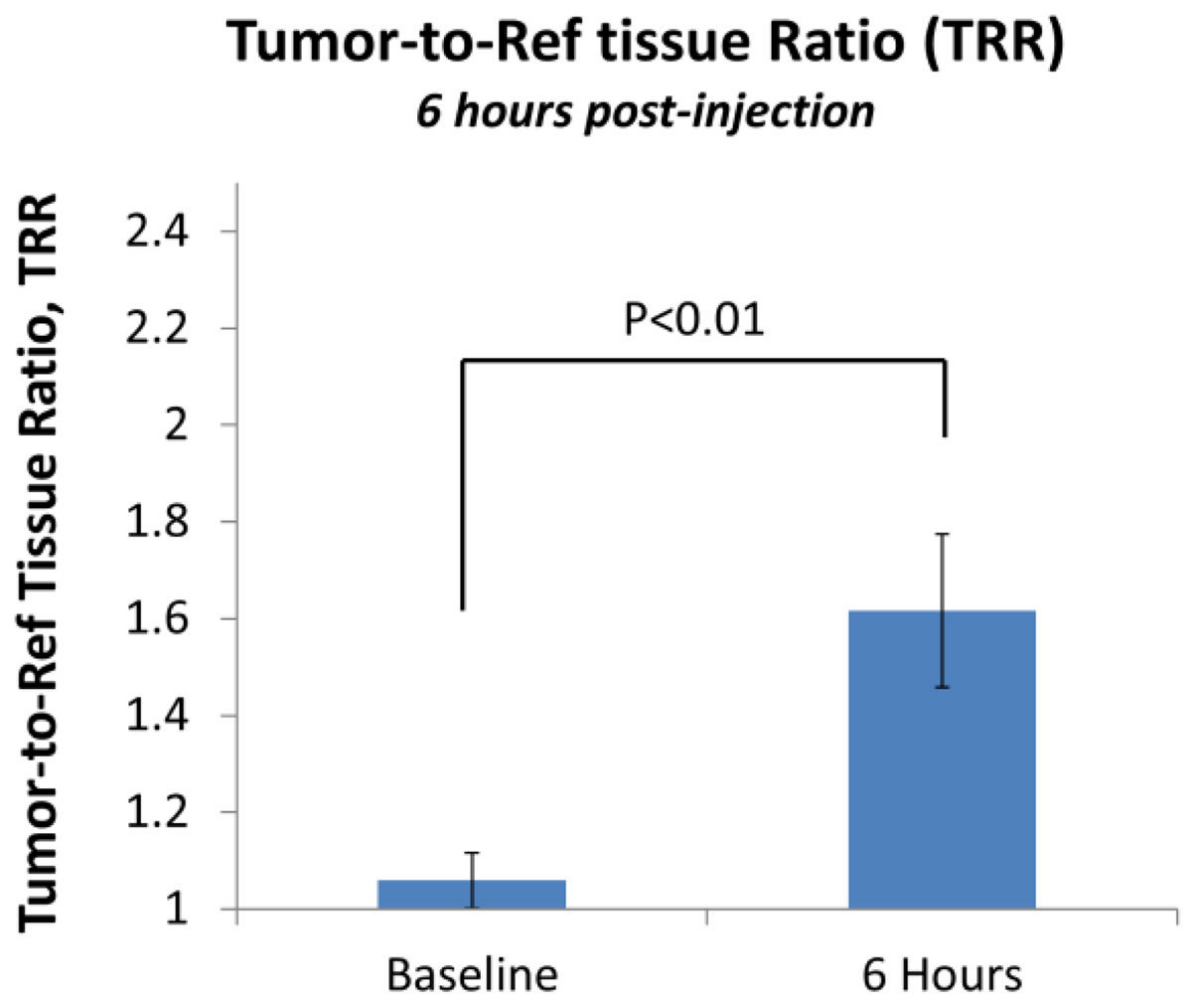
The TRR for a Cohort of Ovarian Cancer Xenograft Mouse Model. The average TRR and its standard deviation (n=3) was calculated using NIR imaging (LB981 NightOWL) prior to and 6 hours post-injection of the BI-10^AF750^ MI probe (80 μg).

**Figure 4:**
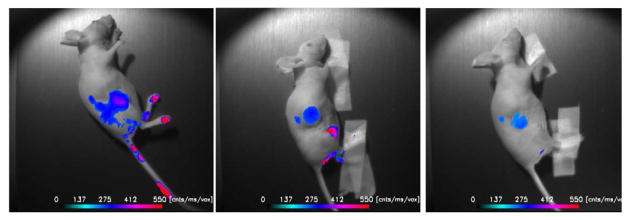
*In vivo* NIRF Imaging of BI-10^AF750^. Three mice were i.v. injected with 80 μg of the BI-10^AF750^ MI probe, and NIR fluorescence images were acquired 6 hours post injection. A threshold was applied to better depict the tumors. Signals in the kidneys and bladder indicate renal filtration. No signal was observed in the liver at this time. An indication of tumor cell growth along the subcutaneous needle injection was observed in two mice (*left* and *right*).

**Figure 5:**
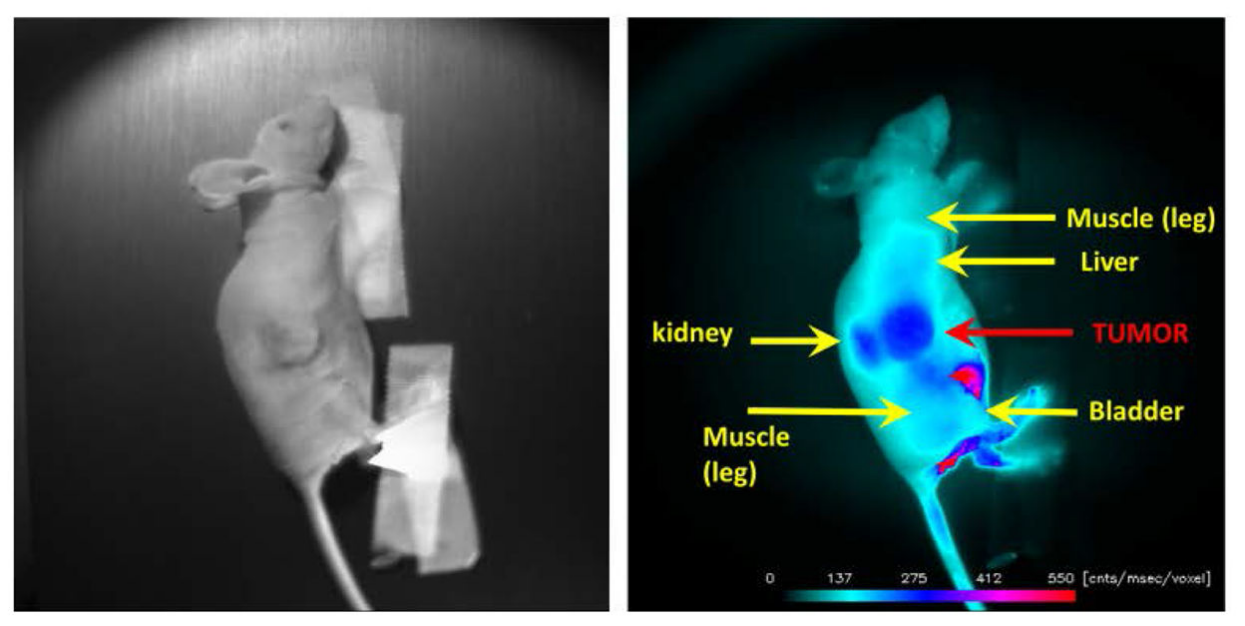
NIR Optical Images of BI-10^AF750^ Molecular Imaging (MI) Probe. *(Left)* Displayed is the bright field image of a mouse with an ovarian tumor on its flank, and *(Right)* the corresponding fluorescence image 6 hours post-injection of the BI-10^AF750^ MI probe. The arrows depict the anatomical features of the mouse.

Displayed in Figure 2 are plots of the BI-10^AF750^ pharmacokinetics. In Figure 2, the tumor-to-reference tissue signal (TRR) is plotted as a function of time for the following doses of BI-10^AF750^–120 μg (M1, blue diamonds), 40 μg (M2, green triangles), and 15 μg (M3, red squares). From this data, the peak TRR signal occurs at approximately 5–6 hours post injection, while the washout rate determined from the average dose-normalized reference fluorescence signal (Figure 2) is approximately 4 hours post injection. For the mice in this second group, the average value of TRR (for M4-M6) for images acquired six hours post injection is 1.62 ± 0.16, which is significantly larger to baseline measurements of 1.05 ± 0.05 (P<0.01; two-tailed t-test) (Figure 3). The reproducibility of the fluorescent signal was at nearly equal levels in all three tumors (Figure 4), where a TRR of approximately 10% was calculated. Signals observed in the kidneys and bladder indicates renal filtration and excretion, with no signal observable signal detected in the liver at this time (Figure 5).

## Discussion

Even though ovarian cancer (OC) is the eighth most common cancer, it causes more deaths compared to all other female gynecological cancers (CDC). Because specific symptoms associated with OC occur late in disease progression, 75 percent of all cases are diagnosed at stage III or IV resulting in a 5 year survival rate of 5 percent. Identifying a select set of biomarkers capable of covering the molecular heterogeneity of OC can help improve the early detection and debulking of ovarian cancer, detection of residual or recurrent disease, the staging and planning of therapy, and enhance targeted therapeutic interventions. When developing, screening and implementing new imaging agents prior to clinical translation, optical imaging provides an efficient set of *in vitro* and *in vivo* assays while avoiding the use of ionizing radiation and costs associated with nuclear medicine techniques (equipment, space, and maintenance) associated with PET and SPECT. OctreoScan is a successful example where many peptide compounds are tested in early clinical trials [33–37]. In this study, a small peptide (BI-10) antagonist specific to the FSH receptor was synthesized and conjugated to optical and NIR fluorochromes, and used to determine their binding affinity (ED50) to epithelial OC cells and investigate their pharmacokinetics and *in vivo* imaging properties, including the time at which TRR is at maximum binding and uptake and the sensitivity to detect OC in a cohort of mice to investigate disease diagnosis and progression.

To date, three biomarkers as measured in blood plasma or serum are approved by the FDA to screen for ovarian cancer: CA125, HE4, and MSLN. CA125 antigen is a membrane-associated mucin expressed on surface cells undergoing metaplastic differentiation and released within blood plasma. However, CA125 tests are not always recommended. Elevated CA125 levels are observed in only 50% of early stage patients and in many benign and non-gynecological conditions, thus limiting its sensitivity and specificity [38,39]. HE4, human epididymis protein 4, and MSLN, mesothlin, are approved for early stage detection of OC and its recurrence. HE4 is expressed in nearly 90% of serous carcinomas, with little or no expression for the above benign conditions. MSLN is associated with cell adhesion and metastasis and expressed in the early stages of OC. These biomarkers are also expressed on other cancers, including lung, endometrium adenocarcinomas, and pancreatic [39], and thus not specific to OC. The combination of CA125 with HE4 or MSLN has been shown to significantly increase the specificity and specificity in detecting OC compared to CA125 alone. Of these three biomarkers, radiolabeled antibodies against CA125 antigen (OC125) and mucin (TAG-72) has been developed and tested in ovarian cancer patients. Based on clinical studies, the reported sensitivity and specificity for these two tracers was 80–93% and 50–75%, and 68% and 55%, respectively [40–42]. Other disadvantages of these tracers include the reporting of false-positives from nonadencarcinomatous malignancy, benign neoplasms and inflammatory tissue, and the poor pharmacokinetics of these antibody-based ligands where the optimal contrast-to-noise occurred 24–48 hours after injection. To overcome these deficiencies, a small peptide imaging agent specific to the FSH receptor (FSHR) was synthesized and investigated.

The follicle stimulating hormone receptor (FSHR) was chosen as a potential target because of its specificity to OC and overexpression in early-stage ovarian cancer cells and malignancy, where it has been shown to exert mitogenic effects and cell proliferation and associated with cancer stem cell properties [43,44]. Antagonists to FSHR activation have been postulated to enhance therapeutic efficacy by affecting disease progression, angiogenesis, and immune response [45,46]. Therefore, identifying molecular imaging agents capable of localizing FSHR expression in patients with OC would have strong diagnostic and therapeutic value. The deca peptide (BI-10) derived from FSH-BI and conjugated with near-infrared fluorescence dye was investigated and shown to bind to ovarian cancer cells *in vivo* with low binding affinit*y* and repeatable differential binding to ovarian tumors in mice.

BI-10^FAM^ and BI-10^AF750^ have a number of advantages as *in vivo* molecular imaging agents, such as its small size, specificity to FSHR (uninhibited binding of hGC), internalization via endocytosis pathway, negligible physiologic effects when used in low doses [47], and easier synthesis and conjugation to other molecules for diagnostic and therapeutic purposes [48]. Their small effective hydrodynamic diameter, high washout rates, and rapid renal filtration are highly valued for *in vivo* imaging [49], where an optimal tumor-to-reference tissue ratio (TRR) can be quickly reached. This was demonstrated for BI-10^AF750^, where the maximum TRR occurred within 4–6 hours after injection (Figure 3) due to the high tissue washout rate and rapid renal filtration. These results are consistent with similar sized molecular imaging agents [50] and significantly shorter than their monoclonal antibody counterparts. Repeated experiments of the BI-10^AF750^ probe (80 μg) resulted in preferential surface binding to OC tumor cells (TRR significantly greater than pre-injection value (P<0.01), Figure 5) and a small variation in TRR within a cohort of mice (10 percent) with the same dose and imaging protocol (TRR=1.62 ± 0.16). Based on these results, a minimal cohort size (e.g., five mice) can be used to achieve a significant TRR value (e.g., TRR>1.3; power analysis; α =0.05, β =0.8), and would suggest using 40 μg dose or higher (BI-10^AF750^) for detection studies (dependency on the optical depth of the tumor) and 120 μg for uptake studies (Figure 3).

To quantify BI-10 binding to the FSHR cell surface receptors of OVCAR-3, the EC50 of the BI-10 fluoroscein conjugate (BI-10^FAM^) was measured *in vitro* using flow cytometry to be 160 μM. Fluoroscein was chosen because the flow cytometer could not operate at wavelengths exceeding 635 nm, which excludes AF750. However, the influence of the change in fluorochrome on the ED50 is not expected to significantly change given that the bio conjugation chemistry was the same and the charge and hydrophilicity in these dyes (synthesized through sulfonation) are similar. Compared to the radio-ligand binding assay, Lee et al reported an EC50 of another FSHR-rich Sertoli cells from Bovine Calf testes to be approximately 300 μM [29]. Various BI-10 incubation times in two different FSHR-rich cell lines as well as washing procedures to remove unbound ligands in two different assays mainly contribute the EC50 value estimation from binding curve [29,51]. Although the binding measurement methodology and cell line selection from Lee and this study were not identical, the BI-10 binding curves show that BI-10 binding to FSHR is highly specific but low in affinity. These results are consistent with the observed dose-dependent tumor binding (4–6 hours; TRR=1.96, 1.59 1.39 for 120 μg, 40 μg, and 15 μg, respectively) and uptake (48 hours: TRR=1.44, 1.29, and 1.14 for 120 μg, 40 μg, and 15 μg, respectively), where the relatively low tumor uptake (48 hours) compared to surface binding peak (6 hours) is believed to be due to the peptide’s high dissociation rate [52,53].

To enhance the binding affinity and cellular uptake, multimeric and polymeric constructs can be synthesized by linking monomeric ligands with poly (ethylene glycol) lipids (PEG) [52,54,55]. Radiolabeled multimeric peptides for tumor imaging have been shown to significantly increase the binding affinity to α_v_β_3_ integrin and Her2 receptors, the former of which is under investigation for the potential clinical application to detect tumors in early stage [56–58]. Similarly, a polyvalent ligand consisting of multiple BI-10 peptides attached onto a flexible and semi-rigid linker (PEG) can lead to cooperative binding and an increase in receptor rebinding [59], or equivalently, a significant decrease the dissociation rate [53]. To optimize avidity, the length of the linker is designed to approximate the distance between two FSHR binding sites (inversely proportional to the FSHR density) [60,61], where the optimal linker length can be determined from the FSH-receptor density reported in this paper. Based upon the number of receptors and the average diameter of OVCAR-3 (10 μm), the distance between two receptors on membrane is approximately 50 angstrom, which is consistent with other receptor systems, e.g., human melanocrtin receptor 4 [62]. Another second and complementary approach is to link two peptides that can concurrently bind to both the ectodomain (ECD) and trans-membrane domain (BI-10) of the G-protein receptor (intra-bivalency or allosteric/orthosteric dimers), where the former is responsible for specificity and high affinity while the later initiates receptor activation [30]. Other peptides targeting the ECD with high specificity have been reported to have binding affinities in the low micro-molar levels (5–20 times that of BI-10) [63], while non-peptide agonists report EC50 in the low nano-molar range [64]. For these latter compounds, their large size and complex chemical synthesis in addition to their agonist functionality are disadvantages. By combining ECD and trans-membrane peptides onto a multimer construct, a molecular imaging agent with sub micromolar binding affinities is possible.

In recent studies FSHR has been reported to play a role in immunotherapy of OC and tumor angiogenesis. For the latter, FSHR expression in endothelial cells (ECs) has been reported in a wide range of tumors [65,66] and demonstrated two characteristics to enhance therapy and improve therapeutic interventions. First, the FSH receptor is expressed on the luminal surface of the EC, thus allowing for ligand to bind to and internalize within ECs and would provide an effect means to target tumor vasculature in therapy. Second, the FSHR is preferentially expressed on the vasculature in the periphery of the tumor, thus defining the tumor boundary and potentially residual and recurrent disease after surgery or therapy. Identifying a ligand that could differentially bind to OC and EC expressing FSHR would greatly expand the diagnostic and therapeutic applications.

Ongoing research continues to investigate new promising biomarkers for the early detection of ovarian cancer, such as KLK6/7, GSTT1, PRSS8, FOLR1, and ALDH1 [67]. Of these, a ligand targeting the folate receptor alpha (FOLR1 or FR-α) has been developed as a molecular imaging agent and tested *in vivo*. Clinical studies have shown FR-α to be increased in 90–95% of patients with epithelial OC [68], with little normal tissue expression. When conjugated to technecium-99 m or the fluorescence dye FITC (fluorescein isothiocyanate), radioscintigraphic images in mice [69] and intraoperative fluorescence imaging in OC patients [70] demonstrated differential specificity to ovarian cancer for detection and debulking of tumors, respectively. Clinical translation on the use of this targeted ligand remains in the investigation stage.

In summary, an optical molecular imaging agent was developed a based on the BI-10 deca peptide and demonstrated specific binding to FSHR expressed on ovarian cancer *in vitro* and *in vivo* with low affinity. From in vivo experiments in mice, the pharmacokinetics of the BI-10^AF750^ was measured to have a maximum receptor binding TRR of 5–6 hours post-injection. Based on the dose and optimal imaging time, a cohort of mice was imaged to demonstrate reproducibility and viability of TRR to monitor changes in FSHR expression. However, the relative high doses of BI-10^AF750^ and reduced uptake TRR was indicative of reversible binding. Based on the receptor density measurement from this study, a multimeric/polyvalent construct is outlined to enhance binding affinity, which can be tested in future studies. These studies will determine the viability of this new molecular imaging agent for future preclinical and clinical studies in the detection and targeted therapy of ovarian cancer.

## Supplementary Material

Supplementary figure

## Abbreviations

FSH: Follicle-Stimulating Hormone
FSHR: Follicle-Stimulating Hormone Receptor
OC: Ovarian Cancer
OEC: Ovarian Epithelial Cancer
TRR: Tumor-to-Reference signal Ratio
BI-10: FSHR Binding Inhibitor amino acid fragment
BI-10^AF750^: BI-10 peptide conjugated with near-infrared fluorochrome AF750
BI-10^FAM^: BI-10 peptide conjugated with near-infrared fluorochrome FAM
