# Supplementary material for: Development of Follicle-Stimulating Hormone Receptor Binding Probes to Image Ovarian Xenografts
Source: J Biotechnol Biomater. Author manuscript; Available in PMC 2016 Jan 14. (PMC4712933; doi:10.4172/2155-952X.1000198)
Supplement: Supplementary figure [file NIHMS735508-supplement-Supplementary_figure.pdf]

Supplemental Data

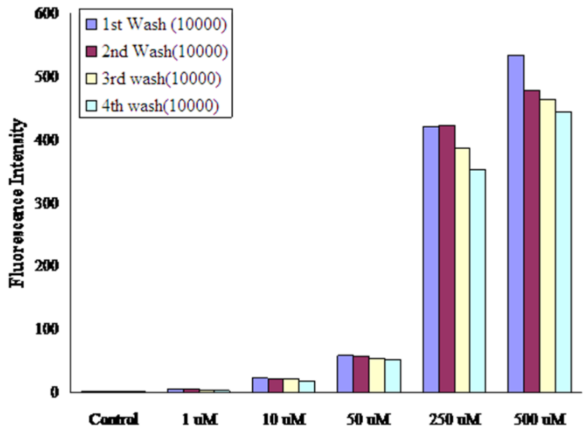

Plotted is the average FITC fluorescence intensity as measure by flow cytometry as a function of the concentration and the number of washes (using PBS). Each data point represents the median fluorescence intensity from ten thousand OVCAR-3 cells after each wash procedure in order to remove unbound peptide. Incubation time of the cells within the medium containing BI-10FAM was 30 minutes. The control sample did not contain BI-10 peptide.
